# Molecular Mechanisms and Future Therapeutics for Spinocerebellar Ataxia Type 31 (SCA31)

**DOI:** 10.1007/s13311-019-00804-6

**Published:** 2019-11-21

**Authors:** Kinya Ishikawa, Yoshitaka Nagai

**Affiliations:** 1 Department of Neurology and Neurological Science, Graduate School of Medical and Dental Sciences, Tokyo Medical and Dental University, Tokyo, Japan; 2 The Center for Personalized Medicine for Healthy Aging, Tokyo Medical and Dental University, Tokyo, Japan; 3 Department of Neurotherapeutics, Osaka University Graduate School of Medicine, Osaka, Japan; 4 Department of Degenerative Neurological Diseases, National Institute of Neuroscience, National Center of Neurology and Psychiatry, Kodaira, Japan

**Keywords:** Neurodegeneration, Ataxia, non-coding repeat, cerebellum, RNA foci, repeat-associated translation, RNA-binding protein, TDP-43, RNA chaperone, Drosophila

## Abstract

Spinocerebellar ataxia type 31 (SCA31) is one of the autosomal-dominant neurodegenerative disorders that shows progressive cerebellar ataxia as a cardinal symptom. This disease is caused by a 2.5- to 3.8-kb-long complex pentanucleotide repeat containing (TGGAA)_n_, (TAGAA)_n_, (TAAAA)_n_, and (TAAAATAGAA)_n_ in an intron of the gene called *BEAN1* (brain expressed, associated with Nedd4). By comparing various pentanucleotide repeats in this particular locus among control Japanese and Caucasian populations, it was found that (TGGAA)_n_ was the only sequence segregating with SCA31, strongly suggesting the pathogenicity of (TGGAA)_n_. The complex repeat also lies in an intron of another gene, *TK2* (thymidine kinase 2), which is transcribed in the opposite direction, indicating that the complex repeat is bi-directionally transcribed as noncoding repeats. In SCA31 human brains, (UGGAA)_n_, the *BEAN1* transcript of SCA31 mutation was found to form abnormal RNA structures called RNA foci in cerebellar Purkinje cell nuclei. Subsequent RNA pulldown analysis disclosed that (UGGAA)_n_ binds to RNA-binding proteins TDP-43, FUS, and hnRNP A2/B1. In fact, TDP-43 was found to co-localize with RNA foci in human SCA31 Purkinje cells. To dissect the pathogenesis of (UGGAA)_n_ in SCA31, we generated transgenic fly models of SCA31 by overexpressing SCA31 complex pentanucleotide repeats in *Drosophila*. We found that the toxicity of (UGGAA)_n_ is length- and expression level–dependent, and it was dampened by co-expressing TDP-43, FUS, and hnRNP A2/B1. Further investigation revealed that TDP-43 ameliorates (UGGAA)_n_ toxicity by directly fixing the abnormal structure of (UGGAA)_n_. This led us to propose that TDP-43 acts as an RNA chaperone against toxic (UGGAA)_n_. Further research on the role of RNA-binding proteins as RNA chaperones may provide a novel therapeutic strategy for SCA31.

## Pentanucleotide TGGAA Repeat Is Tightly Associated with Spinocerebellar Ataxia Type 31

Spinocerebellar ataxia type 31 (SCA31) is one of a group of disorders that shows progressive cerebellar ataxia as a cardinal symptom with an autosomal dominant inheritance. This disease was first described when one of us (KI) and his colleagues found their families are mapped to a long arm of chromosome 16 [1]. We mapped the causative gene to chromosome 16q22.1 [2], followed by identification of a single-nucleotide exchange (C-to-T) in the 5′ untranslated region of a gene, *PLEKHG4* (also called puratrophin-1) that encodes a protein with a spectrin repeat and Rho guanine-nucleotide exchange-factor domain [3]. However, 2 affected subjects without this single-nucleotide exchange were subsequently found [4, 5], indicating that this is a polymorphism rarely found in Japanese population. In fact, many affected individuals across different families shared many rare variants in the critical 2-megabase chromosomal region. Thus, SCA31 was considered to have a strong founder effect [5]. In support of notion, SCA31 was not found in any other countries except Japan [6, 7]. Fortunately, we were able to narrow down 1 border of the critical region at this C-to-T in *PLEKHG4*, and the other border at rs11640843 with a recombination among affected individuals demonstrating a new 900-kb critical region [5].

**Fig. 1:**
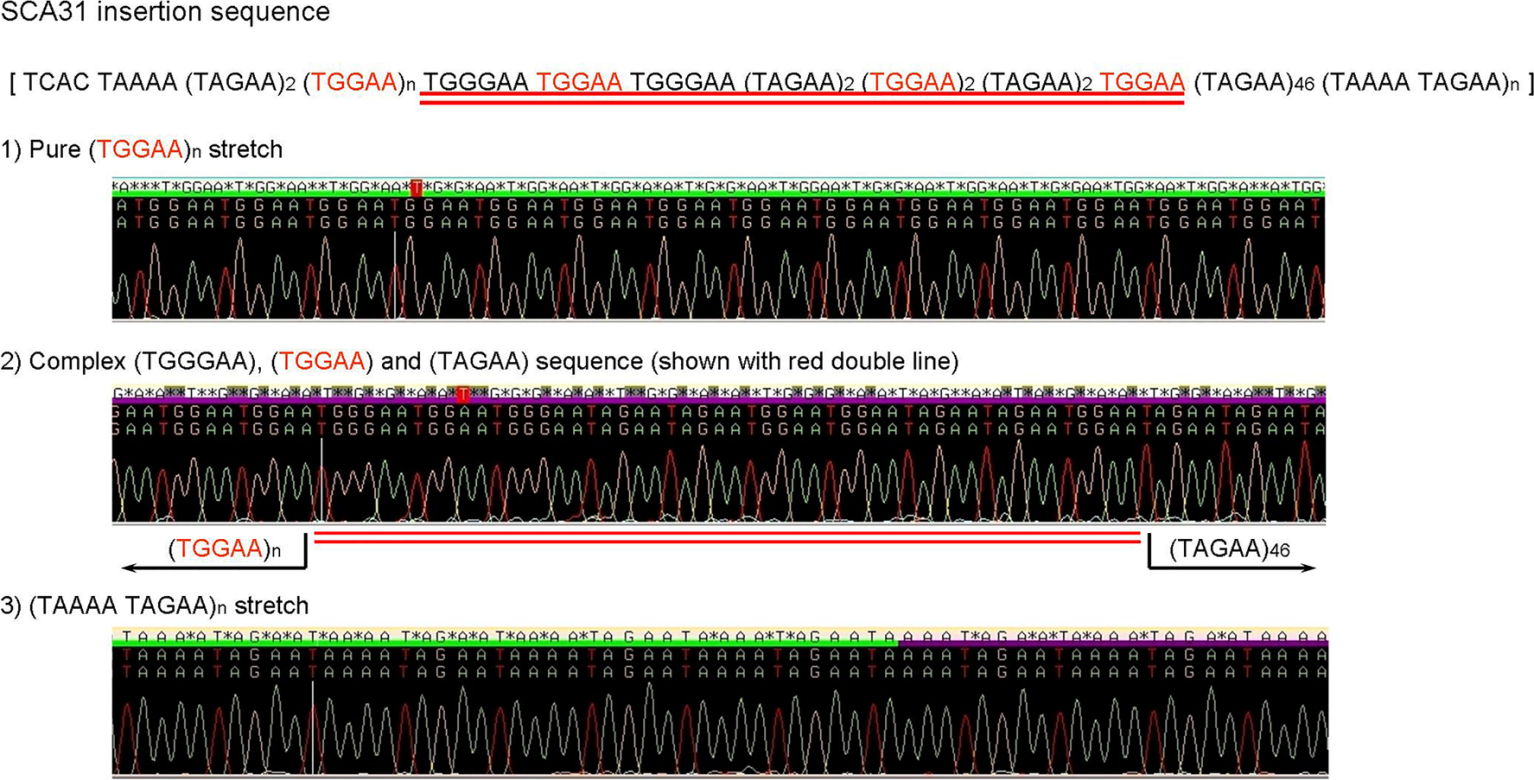
Pentapeptide repeat in the SCA31 locus. Reprinted with permission from American Journal of Human Genetics, 68: 355–367, 2009 [8]

By a tiling search of small rearrangement by Northern blot analysis in conjunction with tiling-path shotgun sequencing of the newly set critical SCA31 chromosomal region in 16q22.1, a mutation shared by all family members with founder chromosomes was found to be a 2.5- to 3.8-kb-long insertion [8]. Cloning and sequencing of this insertion revealed that the internal sequence was a complex pentanucleotide repeat containing (TGGAA)_n_, (TAGAA)_n_, (TAAAA)_n_, and (TAAAATAGAA)_n_ (Fig. 1). The length of this insertion was inversely correlated with the age of onset in SCA31 patients. In contrast, this insertion was not seen in a larger set of control chromosomes with only a few exceptions. The vast majority (99.7%) of Japanese had a short TAAAA repeat of only 8 to 20 repeats. Sequencing the rare, large insertions in control revealed that the internal sequences were different from those in SCA31 subjects: the sequences were either a long pure stretch of (TAAAA)_n_ or a complex repeat with (TAAAA)_n_, (TAGAA)_n_, and (TAAAATAGAA)_n_. The (TGGAA)_n_ was never observed in controls. In addition, presence of TGGAA repeat in SCA31 patients was also replicated in other researchers on different sets of patients [9]. From these observations, (TGGAA)_n_ was the only repeat segregating with the phenotype, suggesting its importance in pathogenesis.

The insertion initially appeared to be unrelated to nearby genes, *BEAN1* (brain expressed, associated with Nedd4) and *TK2* (thymidine kinase 2). However, extensive 3′-RACE experiments revealed that these 2 genes both had multiple downstream exons that had not been deposited in the public databases. This means that the 2.5- to 3.8-kb-long insertion is in an intronic region shared by 2 different genes, *BEAN1* and *TK2* [8]. Although *BEAN1* drives brain-specific expression, *TK2* was expressed in all tissues that we examined. As predicted, the TGGAA repeat was transcribed as UGGAA repeat in SCA31 brains.

Identification of SCA31 repeat clarified clinical picture of SCA31. Typical clinical features can be found in some case reports and cohort studies [10–12]. Sakakibara and her colleagues [10] studied 6 SCA31 patients. Their average age of onset was 63.8 years. When compared with their own SCA6 patients, they found that SCA31 patients’ clinical features were much more confined to cerebellar dysfunction, whereas their SCA6 patients showed pyramidal tract signs and psychiatric features besides cerebellar ataxia. Itaya and her colleagues described 1 SCA31 subject who additionally showed blepharospasm [11]. Their patient developed dysarthria at his age of 56. Clinical examination at his age of 58 revealed slight ataxia of the trunk and lower limbs as well as dysarthria and blepharospasm. Magnetic resonance imaging of his brain revealed cerebellar atrophy most pronounced in the upper vermis, which is typical for SCA31. Nakamura and his colleagues collected 44 patients with SCA31 and underwent a 4-year prospective study [12]. They evaluated patients yearly using the Scale for the Assessment and Rating of Ataxia (SARA) and the Barthel Index (BI). They showed the annual progression of the SARA score was 0.8 ± 0.1 points/year and that of the BI was − 2.3 ± 0.4 points/year (mean ± standard error). Nakamura described that their patients developed ataxic symptoms at 58.5 ± 10.3 years, become wheelchair-bound at 79.4 ± 1.7 years, and died at 88.5 ± 0.7 years. This is the first study to show natural course and disease progression of SCA31.

## Founder Effect in SCA31 and Its Implication

As described, the SCA31 shows a strong founder effect. Although SCA31 is a common ataxia in Japan, this disease is very rare even in neighboring countries such as Korea [7], Taiwan [13], and China [14, 15]. SCA31 was found in Brazilian SCA patients; however, these SCA31 patients were all descendants of Japanese immigrants [16]. In accord with this notion, SCA31 with (TGGAA)_n_ was never found in the Caucasian SCA families (*n* = 320) in French and German cohorts nor in the 588 healthy control subjects [17]. Interestingly, nearly 5.5% of the whole SCA and control groups harbored expansions of different pentanucleotide repeats. The most common repeat was (TACAA)_n_. Other repeats such as (GAAAA)_n_, (TGAAA)_n_, and (TAACA)_n_ were also seen. Unlike the SCA31 insertion consisted of 3 different repeats (TGGAA)_n_, (TAGAA)_n_, and (TAAAA)_n_, the repeats found in Caucasians were all pure stretches. The most common (TACAA)_n_ sometimes showed expansion up to 6.5 kb without clinical manifestation.

This observation indicates 2 things. First, the SCA31 locus in which the vast majority of human beings harbors a short TAAAA repeat (8–20 repeats) can be expanded. The TAAAA can become unstable and expanded as we encountered long (TAAAA)_n_ in Japanese controls. However, all the expanded sequences in Caucasians were all single-nucleotide mutant versions of TAAAA. For example, (TACAA)_n_ is a single A-to-C transition form. Such a mutation may make the SCA31 locus unstable. Second, the TGGAA repeat is the only sequence so far that leads to human disease. Considering that the TAGAA give rise to TGGAA by a single-nucleotide A-to-G transition, it seems likely that the TGGAA repeat, hence SCA31, originates from the TAGAA repeat which has been seen only in Japanese so far.

## Neuropathology of SCA31

**Fig. 2:**
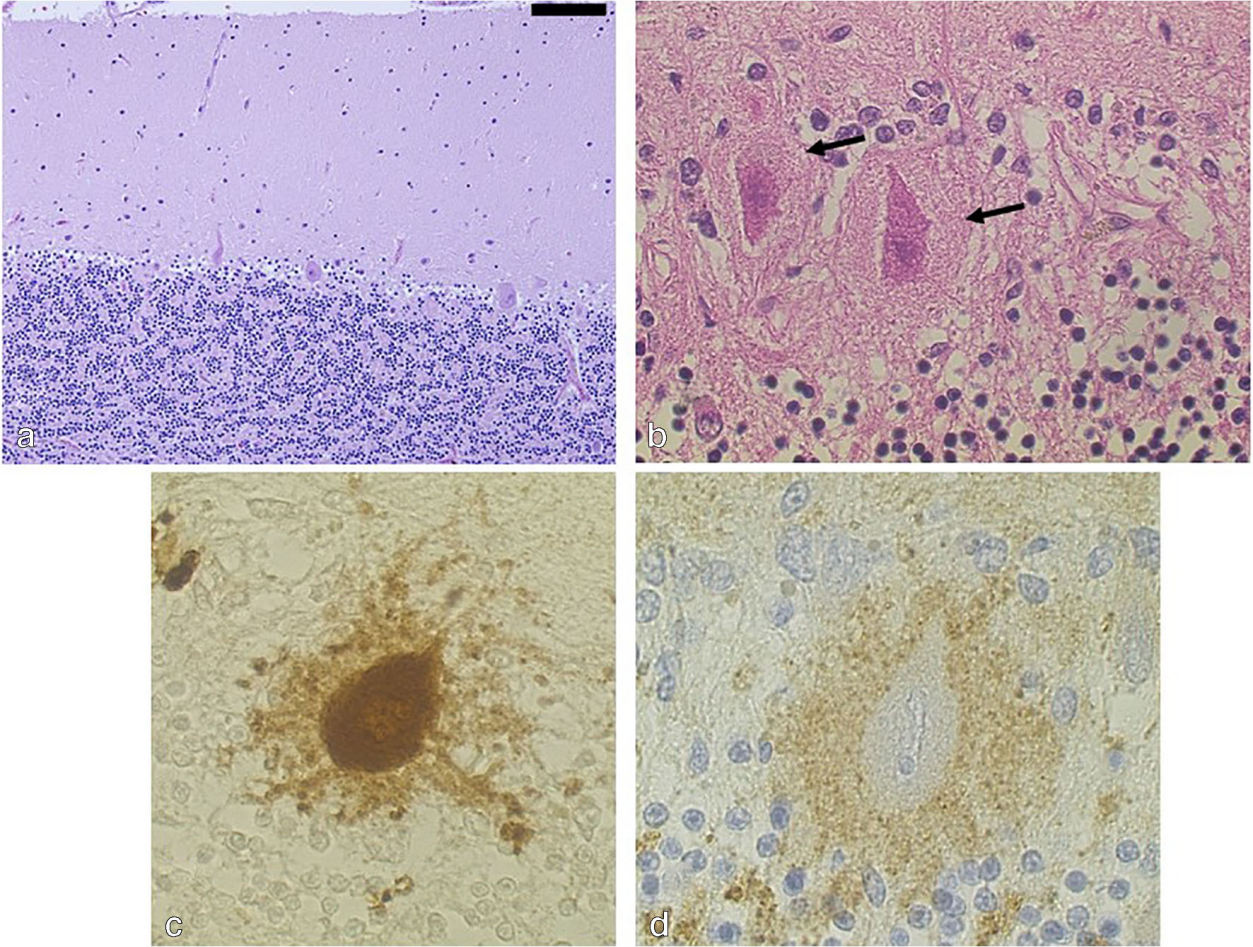
Histopathology of SCA31 patients’ Purkinje cells. (**A**) A moderate Purkinje cell dropout. Bar = 100 μm. (**B**) A Purkinje cell body surrounded by the halo-like amorphous materials (arrow). (**C**) Calbindin-D_28k_ immunohistochemistry. Numerous calbindin-positive somatic sprouts are seen from the Purkinje cell. (**D**) Increased immunoreactivity against synaptophysin, a presynaptic marker protein, surrounding the Purkinje cell. Reprinted with permission from Neurology. 65: 629–632, 2005. [18]

As SCA31 clinically shows progressive ataxia with a purely cerebellar syndrome, and magnetic resonance imaging shows isolated cerebellar atrophy, the neuropathology of SCA31 is expected to be confined to the cerebellar cortex. The first neuropathological study on a 95-year-old female patient who had had SCA31 for 20 years indeed disclosed cerebellar cortical degeneration with Purkinje cell predominant neuronal loss [18] (Fig. 2A). What is very unique to SCA31 was that the Purkinje cell often showed shrinkage of its cell body. In addition, an ill-defined amorphous structure was often seen surrounding the Purkinje cell body [18]. On hematoxylin and eosin staining of the cerebellar tissues, shrunken Purkinje cell body appeared dense pink, whereas the amorphous structure gave pale pink, resembling the halo of the Lewy body in Parkinson’s disease (Fig. 2B). Immunohistochemistry against calbindin-D_28k_, a useful Purkinje cell marker, revealed sprouts form the Purkinje cell body (Fig. 2C), which are infrequently observed in other conditions. The Purkinje cell’s somatic sprout has been described as a pathological hallmark of Menkes’ disease, in which synaptic inputs to the Purkinje cell are known to be dramatically decreased. Although SCA31 is similar to Menkes’ disease in terms of the Purkinje cell’s sprouts, synaptophysin immunohistochemistry disclosed accumulation of presynaptic terminals (Fig. 2D). Thus, the Purkinje cell degeneration in SCA31 appears distinct from that in Menkes’ disease. The structure of the SCA31 Purkinje cell is also different from other ataxias such as SCA6. Ubiquitin-positive inclusions are sometimes seen in the amorphous structure. As this structure was so remarkable, several brain samples with this figure were suspected to be SCA31 and undergone for SCA31 mutation screen, later proven to have SCA31 insertions. One of us (KI) and his mentor, Mizusawa newly coined a word, “halo-like amorphous materials” to this structure as the pathological hallmark of SCA31 [19] (Fig. 2B). As described later in this chapter, the pentapeptide repeat protein specifically translated from the transcript of TGGAA repeat was seen mainly in the amorphous materials.

Yoshida and his colleagues investigated 2 SCA31 brain samples [20]. They not only found the halo-like amorphous materials in their samples, but also found that Purkinje cells surrounded by the amorphous materials tend to show bent, elongated, and often folded nuclei with fragmented Golgi apparatus in the cell soma.

**Fig. 3:**
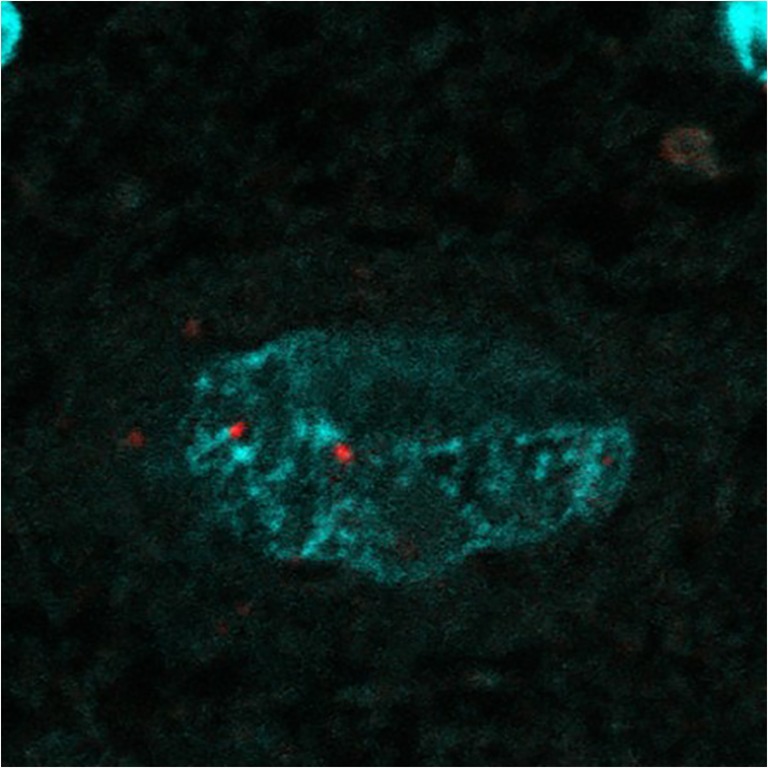
(UGGAA)_n_ containing RNA foci in human SCA31 Purkinje cell. Reprinted with permission from Neuropathology, 33:600–611, 2013. [21]

From the genetic observation, the (TGGAA)_n_ transcribed into (UGGAA)_n_ as BEAN1 transcripts has been implicated as an important factor of SCA31 pathogenesis. To gain further insight into the SCA31 pathogenesis, *in situ* hybridization using RNA probes against (UGGAA)_n_ or (UAGAAUAAAA)_n_ was performed. Yusuke Niimi and his colleagues identified RNA foci within SCA31 Purkinje cells’ nuclei labeled positive with a locked nucleic acid (LNA)-oligonucleotide (TTCCA)_5_ probe [21] (Fig. 3). Similar RNA foci were also detected by probes against (UAGAAUAAAA)_n_ [21]. They also tested whether (UGGAA)_n_ is toxic than (UAGAAUAAAA)_n_ in cultured cells. They created transient and stable expression cell systems and found that cell toxicity and formation of RNA foci were both consistently observed upon the expression of (UGGAA)_n_. These observations led us to conclude that (UGGAA)_n_ could be toxic in cells.

## Animal Models of SCA31 and Their Implication in the Molecular Pathogenesis and Therapies

### Establishment of *Drosophila* Models of SCA31

To elucidate the molecular pathomechanisms of SCA31, establishing animal models is indispensable. We first tried to establish *Drosophila* models of SCA31 to express an expanded (UGGAA)_n_ repeat RNA [22], because *Drosophila* provides a rapid and powerful tool to model human neurodegenerative diseases [23], and *Drosophila* models of SCA8 expressing (CUG)_n_ repeat RNAs have been successfully established [24]. We first subcloned an SCA31-specific expanded (TGGAA)_n_ repeat followed by complex repeats consisting of (TAGAA)_n_, (TAAAA)_n_, and (TAAAATAGAA)_n_, from SCA31 patients, and established transgenic flies expressing expanded (UGGAA)_n_ repeats (UGGAA_exp_) RNA under the *UAS/GAL4* system. We also designed control transgenic flies carrying complex (TAGAA)_n_, (TAAAA)_n_, and (TAAAATAGAA)_n_ repeats that are found in the normal Japanese population.

**Fig. 4:**
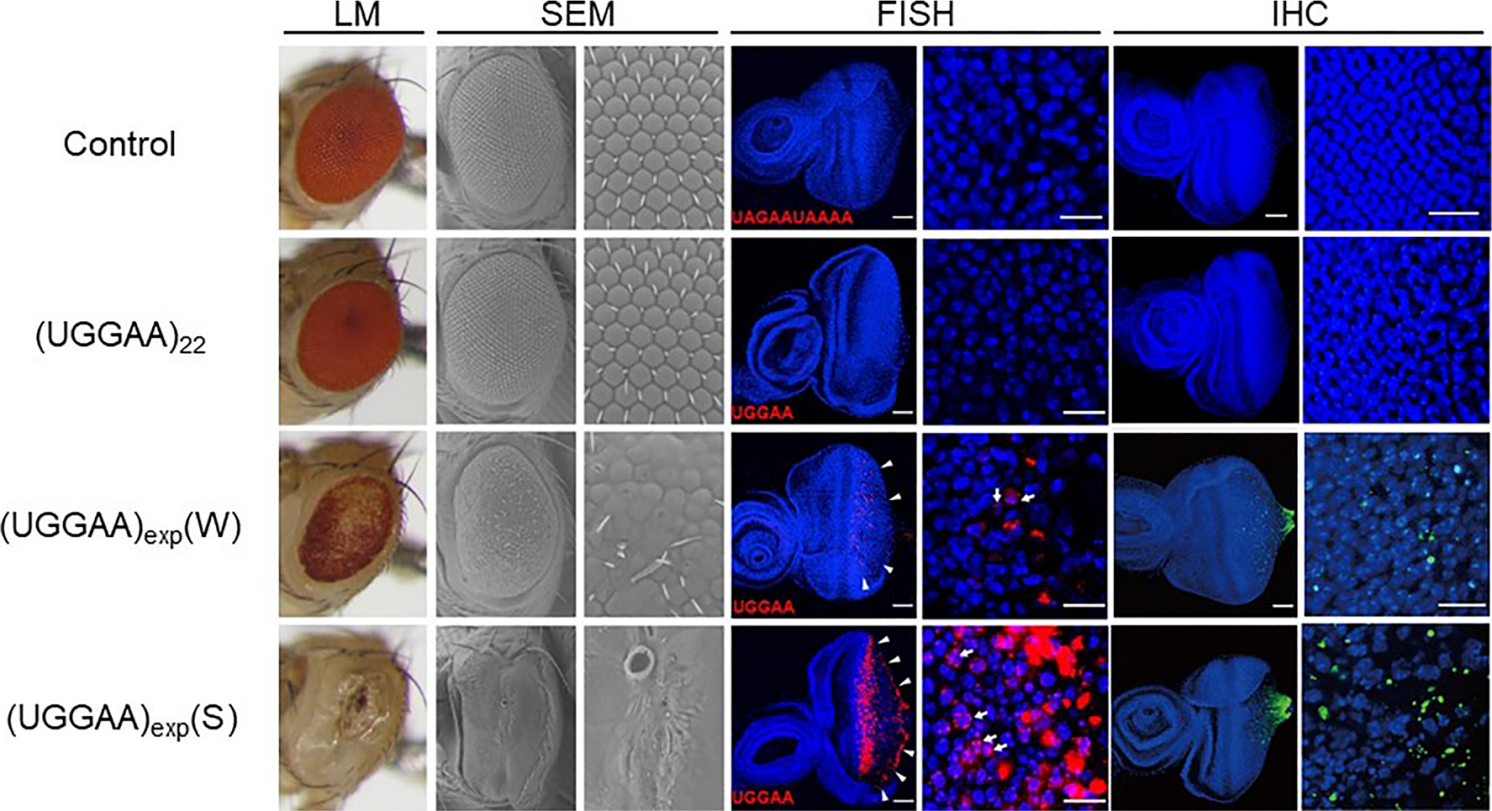
Expression of UGGAA_exp_ RNA causes compound eye degeneration accompanied by RNA foci formation and PPR protein production in Drosophila. Light microscopy (LM) and scanning electron microscopy (SEM) images of compound eyes, and RNA fluorescence *in situ* hybridization (FISH) and immunohistochemistry (IHC) analyses of eye imaginal discs of flies expressing the control repeats, UGGAA22, or UGGAAexp. UGGAAexp(W): weak expression line, UGGAAexp(S): strong expression line. Reprinted with permission from Neuron, 94(1):108–124.e7, 2017 [22]

Upon their expression in the compound eyes of flies using the *GMR-GAL4* driver, we found that UGGAA_exp_ (80–100 repeats) RNA caused remarkable eye degeneration depending on its expression level, whereas control repeat and short UGGAA_22_ RNA, which happened to be generated by spontaneous contraction of the TGGAA repeats, had no significant effect (Fig. 4). We next expressed UGGAA_exp_ in the nervous system after the eclosion using the inducible *elav*-GeneSwitch driver, and found that the expression of UGGAA_exp_ exhibited a shorter lifespan and progressive locomotor defects, while control repeat and UGGAA_22_ did not. RNA fluorescence *in situ* hybridization (FISH) revealed extensive accumulation of UGGAA_exp_ RNA as RNA foci in eye imaginal discs of UGGAA_exp_-expressing flies (Fig. 4), consistent with the pathology of SCA31 patients [18]. We next examined whether pentapeptide repeat (PPR) proteins are produced by the repeat-associated translation from the UGGAA repeat RNA in flies expressing UGGAA_exp_. Indeed, PPR proteins were detected by anti-PPR antibodies in eye imaginal discs of UGGAA_exp_ flies depending on its RNA level, whereas no PPR was detected in the UGGAA_22_ flies (Fig. 4). In human SCA31 cerebellar tissues, we also found PPR protein aggregates mainly in the amorphous materials surrounding the Purkinje cell. From these results, we successfully established *Drosophila* models of SCA31 showing UGGAA_exp_-mediated neurotoxicity accompanied by formation of RNA foci and production of PPR proteins, which faithfully recapitulate the pathological features of SCA31 patients [22].

### Novel Function of TDP-43 as an RNA Chaperone for UGGAA Repeat RNA Aggregation and Repeat-Associated Translation in SCA31 Flies

Considering that proteins sequestered in RNA foci have been reported to play an important role in the pathogenesis of noncoding repeat expansion disorders [25], Sato N. and our colleagues screened for potential UGGAA_exp_-binding proteins by *in vitro* RNA pulldown assay using nuclear fraction of PC12 cells. Similar approach using mouse brain lysates was performed by our co-investigators N. Charlet-Berguerand and C. Sellier. Interestingly, several ALS/FTD-linked RNA-binding proteins (RBPs) were identified as UGGAA-binding proteins, such as TDP-43 (TAR DNA-binding protein, 43 kDa), FUS, and hnRNPs. After confirming TDP-43 in the (UGGAA)_n_-bound protein fraction, we further confirmed co-localization of TDP-43 with RNA foci in human SCA31 Purkinje cells, and thus decided to focus on TDP-43 to analyze its role in the SCA31 pathology [22].

**Fig. 5:**
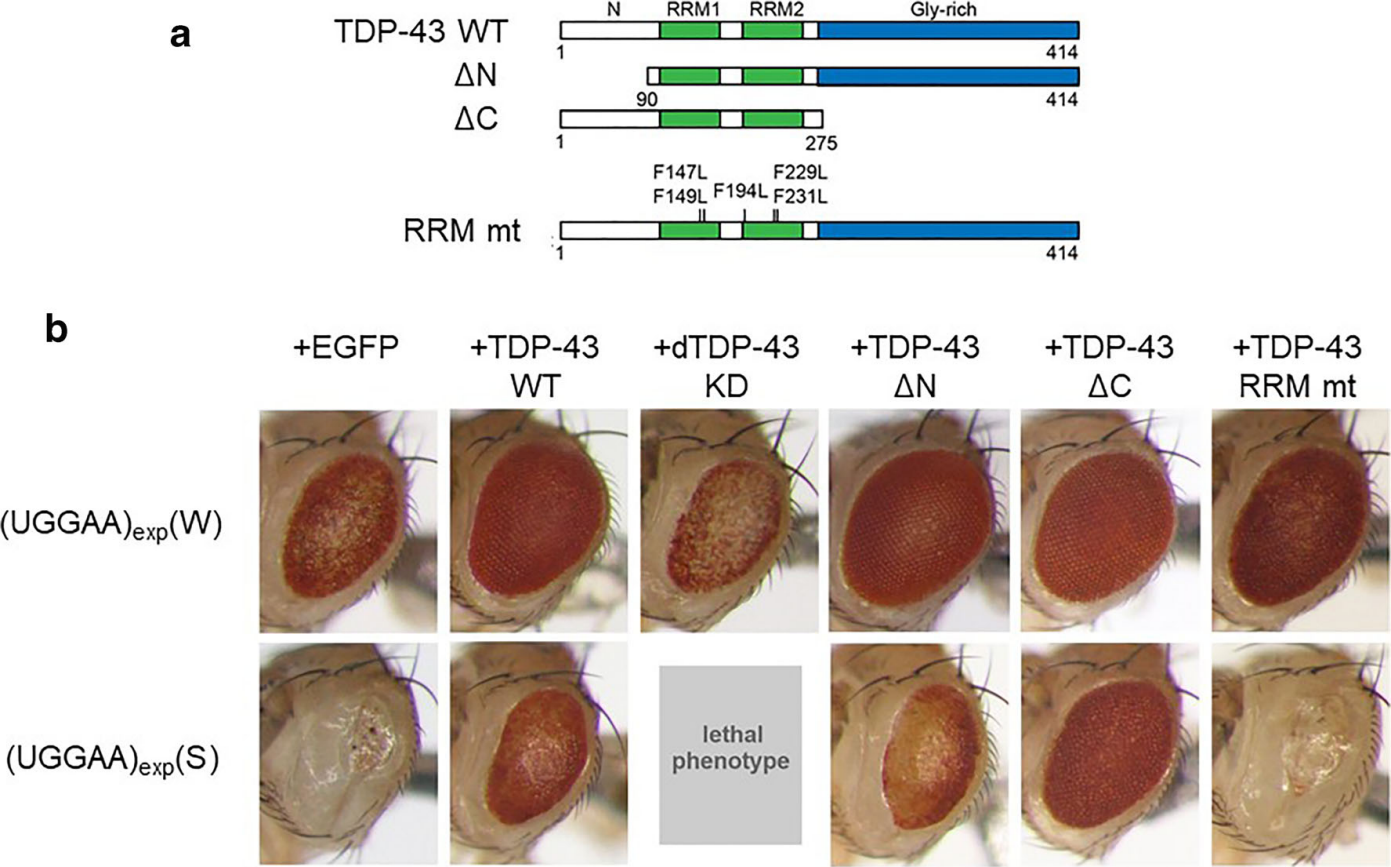
Co-expression of TDP-43 suppresses compound eye degeneration in SCA31 flies expressing UGGAA_exp_ RNA via its RNA-binding ability. (a) Schematic diagram of TDP-43 WT, TDP43 mutants with N- or C-terminus-deletion (DN or DC), or RNA recognition motif (RRM) mutations (RRM mt). (b) LM images of compound eyes of flies coexpressing TDP-43 variants or endogenous dTDP43 RNAi with UGGAAexp. Reprinted with permission from Neuron, 94(1):108–124.e7, 2017 [22]

To explore the potential roles of TDP-43 in UGGAA_exp_-mediated toxicity, we crossed flies expressing UGGAA_exp_ with those expressing human TDP-43. Surprisingly, co-expression of TDP-43 strikingly suppressed compound eye degeneration in the UGGAA_exp_-expressing flies (Fig. 5). In contrast, RNA interference (RNAi)–mediated knockdown of endogenous *Drosophila* TDP-43 significantly enhanced eye degeneration in the UGGAA_exp_ flies, indicating a crucial role of TDP-43 in UGGAA_exp_-mediated toxicity *in vivo* (Fig. 5). However, TDP-43 carrying mutations in the RNA recognition motif (RRM), which has reduced RNA-binding activity (RRM mutant), failed to suppress UGGAA_exp_-mediated eye degeneration, further suggesting that the RNA-binding ability of TDP-43 is essential for its suppression of UGGAA_exp_-mediated toxicity (Fig. 5) [22].

**Fig. 6:**
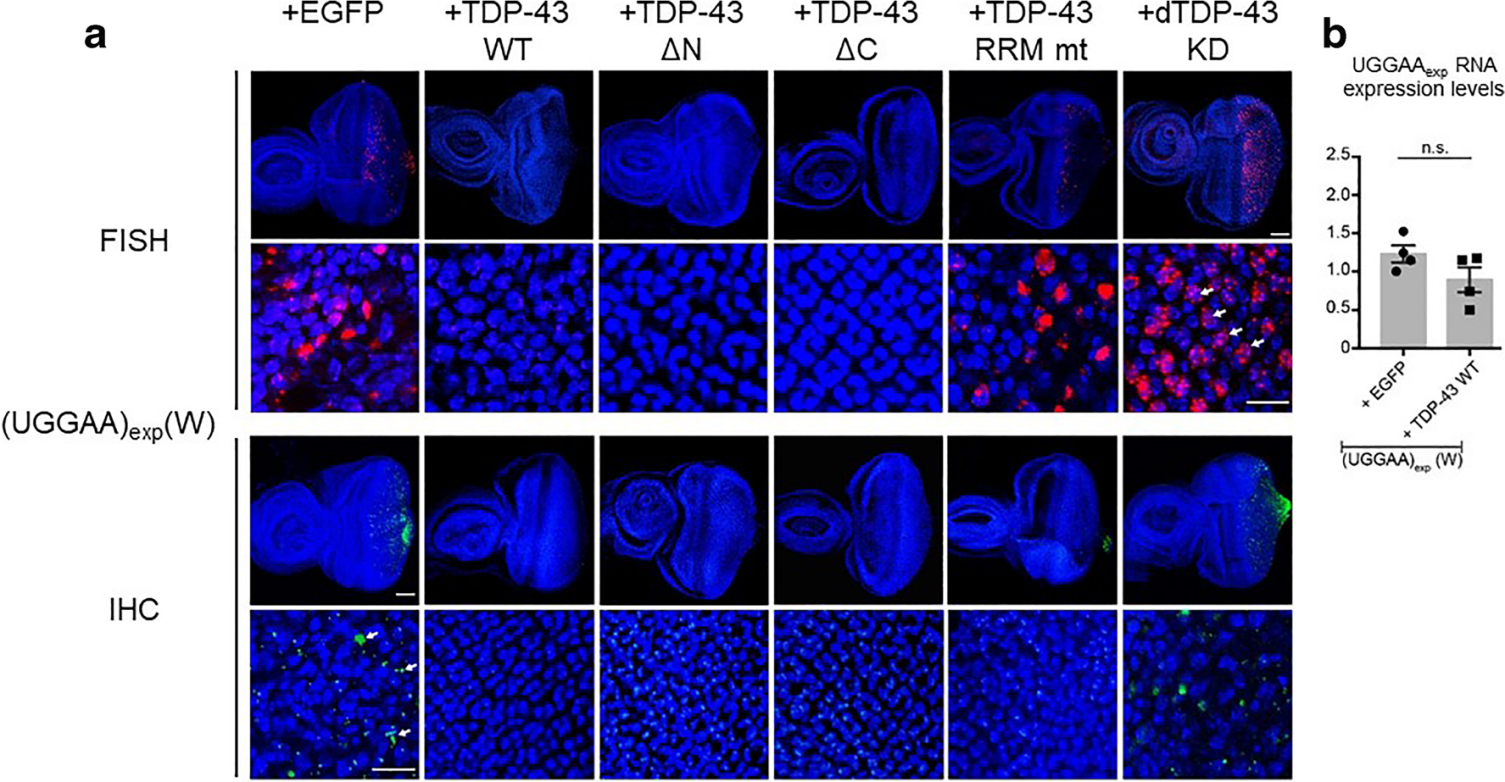
Co-expression of TDP-43 suppresses RNA foci formation and PPR protein production in SCA31 flies expressing UGGAA_exp_ RNA. (a) RNA FISH and IHC analyses of eye imaginal discs of UGGAAexp(W) flies coexpressing TDP-43 variants, or dTDP-43 RNAi. (b) Quantitative real-time PCR analysis of UGGAAexp RNA expression in UGGAAexp(W) flies coexpressing TDP-43. Reprinted with permission from Neuron, 94(1):108-124.e7, 2017 [22]

We next examined RNA foci of UGGAA_exp_ RNA by FISH and found that co-expression of TDP-43 significantly reduced the formation of RNA foci in UGGAA_exp_-expressing flies, whereas co-expression of TDP-43 RRM mutant did not (Fig. 6). RT-PCR analyses confirmed that UGGAA_exp_ RNA expression levels were not altered by co-expression of TDP-43 (Fig. 6), suggesting that TDP-43 does not promote the degradation of UGGAA_exp_ RNA, but rather prevents RNA foci formation, resulting in neutralization of toxic UGGAA_exp_ RNA via their direct interactions. Subsequent circular dichroism spectroscopy (CD) and atomic force microscopy (AFM) analyses were done by our colleagues led by C.E. Pearson demonstrated that the binding of TDP-43 to UGGAA repeat RNA alters its structure and prevents its aggregation, indicating that TDP-43 functions as an RNA chaperone for UGGAA repeat RNA [22, 26]. We also examined the repeat-associated translation and found that co-expression of TDP-43 significantly reduced the production of PPR proteins in UGGAA_exp_-expressing flies (Fig. 6).

We also asked whether other UGGAA_exp_-binding RBPs function as an RNA chaperone for UGGAA repeat RNA, as does TDP-43. Co-expression of FUS or hnRNPA2B1 dramatically suppressed compound eye degeneration in UGGAA_exp_-expressing flies, as TDP-43 did. Moreover, both FUS and hnRNPA2B1 attenuated the accumulation of RNA foci and the production of PPR proteins without affecting the UGGAA_exp_ RNA expression levels, confirming their functions as RNA chaperones [22]. In contrast, molecular chaperones, such as HSP70 or HSP40, did not affect the eye degeneration in UGGAA_exp_-expressing flies. Considering that similar therapeutic effects of other RBPs on noncoding repeat expansion disorders have been reported, such as MBNL1 for both CUG repeat in DM1 [27] and CCUG repeat in DM2 [28], hnRNPA2/B1 for CGG repeat in FXTAS [29], we speculate that these RBPs might also function as RNA chaperones to alter the repeat RNA structure.

### UGGAA Repeat RNA Conversely Suppresses ALS/FTD-Linked RBP Toxicity

**Fig. 7:**
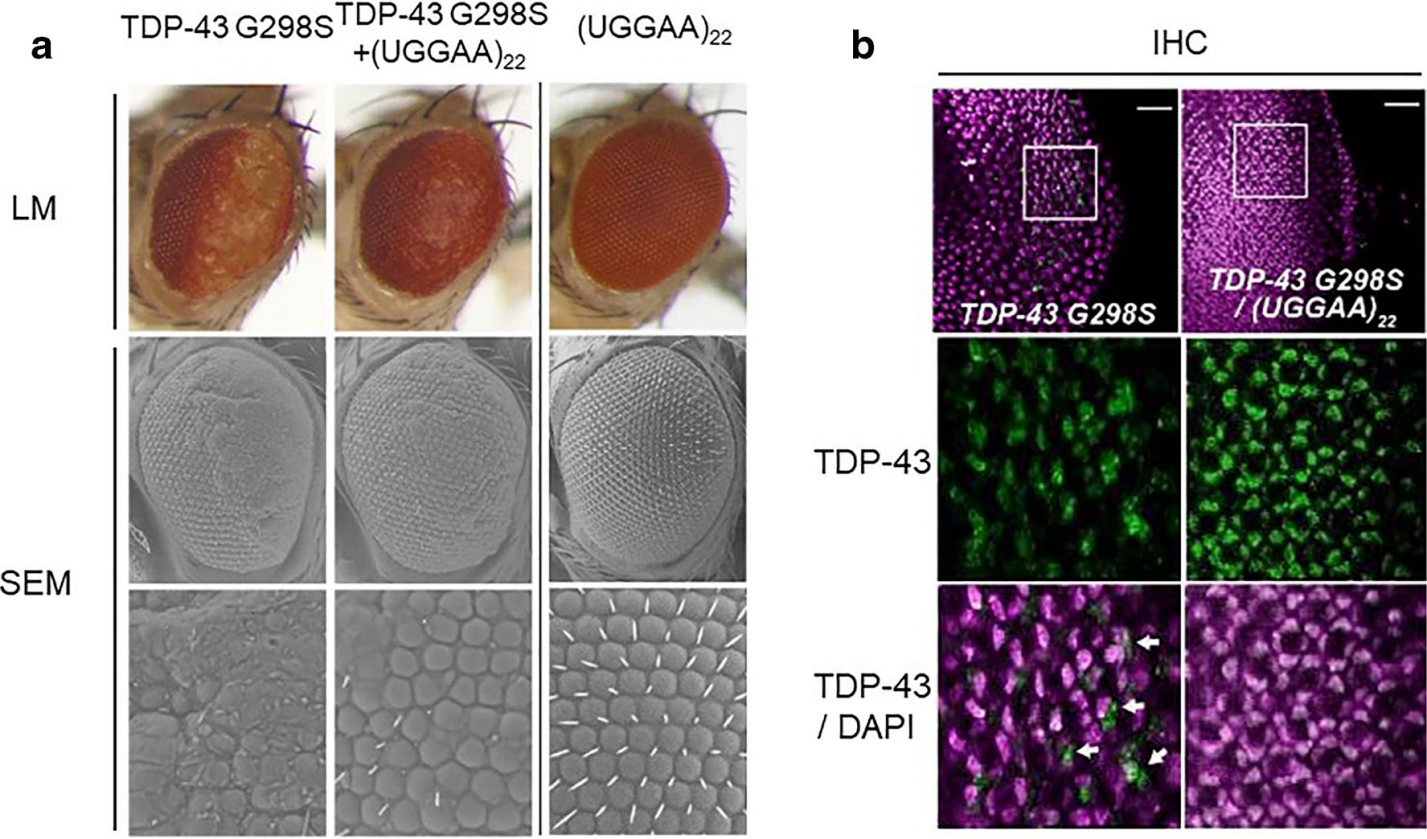
Co-expression of UGGAA_22_ RNA suppresses TDP-43 aggregation and compound eye degeneration in ALS flies expressing TDP-43 G298S. (a) LM and SEM images of compound eyes of flies expressing TDP-43 G298S mutant, UGGAA22, and TDP-43 G298S mutant with UGGAA22. (b) IHC analyses of eye imaginal discs of TDP-43 G298S flies coexpressing UGGAA22. Reprinted with permission from Neuron, 94(1):108-124.e7, 2017 [22]

Our studies highlighted a novel role of TDP-43, FUS, and hnRNPA2B1 as RNA chaperones for UGGAA_exp_ repeat RNA. On the other hand, aggregations of these RBPs are involved in the pathogenesis of ALS/FTD [30]. Overexpression of TDP-43 in experimental animals has been shown to cause neurodegeneration accompanied by its aggregation [31]. Given that the UGGAA repeat binds with these RBPs, it is possible that RBP toxicity may be ameliorated by binding RNAs. To verify this hypothesis, we crossed flies expressing ALS-linked mutant TDP-43 G298S with the UGGAA_22_ flies, which do not show any toxicity. Although TDP-43 G298S-expressing flies exhibited considerable compound eye degeneration, co-expression of UGGAA_22_ significantly suppressed the eye degeneration as well as mutant TDP-43 aggregation (Fig. 7) [22]. This result is consistent with previous reports showing that UG repeat RNA or CLIP34nt RNA which bind to TDP-43 inhibit its aggregation *in vitro* [32, 33]. We also confirmed that co-expression of UGGAA_22_ also suppressed compound eye degeneration of flies expressing either FUS or mutant hnRNPA2B1 D290V.

**Fig. 8:**
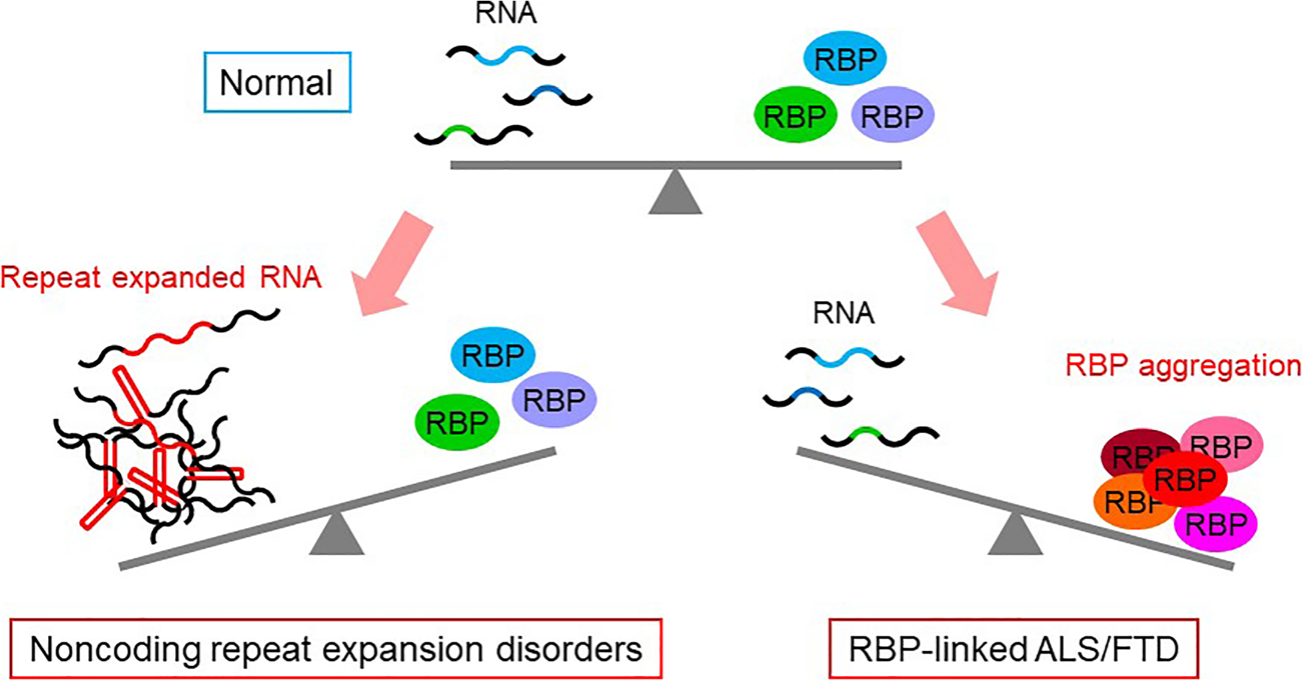
Imbalance between RNA and RBP homeostasis in noncoding repeat expansion disorders and ALS/FTD

These results reveal that functional cross-talk of the RNA-RBP network regulates their own quality and homeostasis, suggesting that balancing the RNA-RBP cross-talk is a potential therapeutic approach for both noncoding repeat expansion disorders and RBP proteinopathies (Fig. 8) [22]. Our hypothesis that imbalance in the RNA-RBP cross-talk leads to their aggregation is further supported by a recent study demonstrating that RNA buffers the phase separation of RBPs [34].

## Perspectives

Currently, there is no fundamental treatment for SCA31. Therefore, RBP may provide a new idea for SCA31 therapeutics. Toward further elucidating the pathomechanisms of SCA31 and developing its potential therapies, we are now developing mouse models of SCA31. If we indeed see RNA foci containing (UGGAA)_n_ in mouse Purkinje cells, immediate need would be to validate the effect of RBP such as TDP-43 in the mouse Purkinje cell degeneration. Given that the RNA-RBP cross-talk is the case in SCA31 mouse models and also in humans, (UGGAA)_n_-binding peptide may dampen its toxicity, which may further lead to a development of SCA31 therapeutics. Finally, disturbance of RNA-RBP cross-talk may not be the only possible mechanism of SCA31 pathogenesis. Further studies would be needed to discover mechanisms that (TGGAA)_n_ leads to Purkinje cell predominant neurodegeneration.
